# Microbiome specificity and fluxes between two distant plant taxa in Iberian forests

**DOI:** 10.1186/s40793-023-00520-x

**Published:** 2023-07-22

**Authors:** Zaki Saati-Santamaría, Rocío Vicentefranqueira, Miroslav Kolařik, Raúl Rivas, Paula García-Fraile

**Affiliations:** 1 Departamento de Microbiología y Genética, Universidad de Salamanca, 37007 Salamanca, Spain; 2 Institute for Agribiotechnology Research (CIALE), Villamayor, 37185 Salamanca, Spain; 3 Institute of Microbiology of the Czech Academy of Sciences, Vídeňská 1083, 142 20 Prague, Czech Republic; 4 Associated Research Unit of Plant-Microorganism Interaction, USAL-CSIC (IRNASA), 37008 Salamanca, Spain

**Keywords:** Plant microbiome, Host-microbe interactions, Blueberry, Blackberry, Soil communities, Rhizosphere

## Abstract

**Background:**

Plant-associated microbial communities play important roles in host nutrition, development and defence. In particular, the microbes living within internal plant tissues can affect plant metabolism in a more intimate way. Understanding the factors that shape plant microbial composition and discovering enriched microbes within endophytic compartments would thus be valuable to gain knowledge on potential plant–microbial coevolutions. However, these interactions are usually studied through reductionist approaches (in vitro models or crop controlled systems). Here, we investigate these ecological factors in wild forest niches using proximally located plants from two distant taxa (blueberry and blackberry) as a model.

**Results:**

Although the microbial communities were quite similar in both plants, we found that sampling site had a high influence on them; specifically, its impact on the rhizosphere communities was higher than that on the roots. Plant species and sample type (root vs. rhizosphere) affected the bacterial communities more than the fungal communities. For instance, Xanthobacteraceae and Helotiales taxa were more enriched in roots, while the abundance of Gemmatimonadetes was higher in rhizospheres. Acidobacteria abundance within the endosphere of blueberry was similar to that in soil. Several taxa were significantly associated with either blackberry or blueberry samples regardless of the sampling site. For instance, we found a significant endospheric enrichment of *Nevskia* in blueberry and of *Sphingobium*, *Novosphingobium* and *Steroidobacter* in blackberry.

**Conclusions:**

There are selective enrichment and exclusion processes in the roots of plants that shapes a differential composition between plant species and sample types (root endosphere—rhizosphere). The special enrichment of some microbial taxa in each plant species might suggest the presence of ancient selection and/or speciation processes and might imply specific symbiosis. The selection of fungi by the host is more pronounced when considering the fungal trait rather than the taxonomy. This work helps to understand plant–microbial interactions in natural ecosystems and the microbiome features of plants.

**Supplementary Information:**

The online version contains supplementary material available at 10.1186/s40793-023-00520-x.

## Background

The ongoing coevolution of microbes and plants has led to beneficial specific interkingdom interactions [1–4]. This is clearly manifested in the plant roots, where, comparing to the rhizospheric soil, the specificity is increased in the endospheric compartments for bacteria and to a lesser extent for fungi [5–7]. The evolutionary success of these interactions over millions of years has led wild plants to ideally adapt to their native environments by taking advantage of their symbionts and vice versa [8–10]. However, beyond the study of nodule-forming symbioses between legumes and rhizobia [11–13] and of the functions of mycorrhizal fungi [14, 15], knowledge of the different plant‒microbe interactions is still scarce [16]. New research on the composition of native microbial communities is needed to identify specific plant‒microbe connections [17].

Despite the existence of specific microbial symbiosis in plants, there are countless factors that can modify the occurrence and proportion of microbes in the plant environment. Among them, the composition of prokaryotes, especially bacteria, is supposed to be more strongly influenced by the plant species and tissue type (rhizosphere, root, shoot or leaves) than the fungal communities, which are mainly affected by climatic conditions and nutrient availability, such as the C/N ratio [6, 17–19]. Hence, the study of plant microbiomes considering spatial scaling is of utmost importance to draw correct conclusions and to unveil plant‒microbe coadaptation patterns [17].

Blueberry (*Vaccinium myrtillus*) and blackberry (*Rubus ulmifolius*) are considered functional foods with rich nutritional value, and thus, these berries are becoming increasingly popular among consumers, which implies a strong economic impact and development prospects [20, 21]. In contrast to blackberry, wild blueberry plants are restricted to acidic soils such as those of forest ecosystems [22]. Despite the importance of these plants, there is no research on the microbial communities of blackberry plants, and the few studies on blueberry are restricted to agricultural fields, only to rhizosphere samples or to some agricultural plant varieties [23–27].

Here, we aim to unravel specific microbial associations with plants. As a model, we used two distant plant taxa (*V. myrtillus* and *R. ulmifolius*) cooccurring along different wild forest ecosystems in the Iberian Peninsula. We searched for specific enrichments or exclusions of microbial taxa in the endospheric tissues to unravel the selective filter that differentiates the rhizospheric and root microbiomes in these plants. Our comparative microbiome study provides new evidence of the ecological shifts between the root and rhizosphere microbial communities as well as new data on the microbiome assembly of blackberry and blueberry plants.

## Methods

### Obtaining samples

We obtained root and rhizosphere samples from wild blackberry (*R. ulmifolius*) (at the end of the flowering stage) and blueberry (*V. myrtillus*) plants from three different locations in the Iberian Peninsula (July-2020); blackberry plants were also collected in a fourth location (Additional file 1: Fig. S1). The plants were collected from forest ecosystems ranging in altitude from 1,015 m to 1,726 m. Forty-two samples were collected in total (3 root and 3 rhizosphere samples from each plant species in each location). To get the rhizospheric soil we shook the roots vigorously within sterile plastic receipts. We measured the pH of the surrounding bulk soil in each location (Additional file 1: Fig. S1). All samples were transported to the laboratory on ice and then immediately stored at -80 °C until processing. Rhizosphere samples were kept frozen until DNA extraction. To process the plants, roots were separated and thoroughly washed with tap water to remove the rest of the soil and dead leaves. Each sample was then washed with sterile distilled water six times with shaking every time to remove all surface microbes. After processing, each sample was immediately stored at -80 °C until DNA extraction.

### DNA extraction and targeted amplicon sequencing

Total genomic DNA was isolated from all samples using the DNeasy PowerSoil kit (Qiagen) according to the manufacturer’s instructions (100 μL for the final elution volume). DNA was quantified using the Qubit High Sensitivity dsDNA Assay (Thermo Fisher Scientific).

For fungal library preparation, a fragment of the ITS genomic region was amplified using the primers ITS5 (5′ GGA AGT AAA AGT CGT AAC AAG G 3′) and ITS2 ngs (5′ TTY RCK RCG TTC TTC ATC G 3′) [28]. A blocking primer set was also used to prevent amplification of the plant DNA. The blocking primers used were ITS1catta_RubusBP (5′ GAT CAT TGT CGA AAC CTG CCC AGC AG 3′) and ITS1catta_VacciniumBP (5′ GAT CAT TGT CGA AAA CCT GCC AAG CAG 3′). Likewise, for bacterial library preparation, the V3–V4 region of the 16S rRNA gene was amplified using the primers Bakt341F (5′ CCT ACG GGN GGC WGC AG 3′) and Bakt805R (5′ GAC TAC HVG GGT ATC TAA TCC 3′) [29]. The blocking primers used in this case were Bakt805R_mitoRubusBP (5′ CTA ATC CCG TTC GCT CCC CAT GCT TTC GCA CTC 3′), Bakt805R_mitoVacciniumBP (5′ CTA ATC CCG TTC GCT CCC CAT GCT TTC GCA CCC 3′), Bakt805R_plastidRubusBP (5′ CTA ATC CCA TTT GCT CCC CTA GCT TTC GTC TC 3′), and Bakt805R_plastidVacciniumBP (5′ CTA ATC CCG CTC GCT CCC CTA GCT TTC GTC TC 3′), which were specifically designed to target either plastidial or mitochondrial DNA of either *Vaccinium* sp. or *Rubus* sp.

Illumina sequencing primers were attached to the PCR primers at their 5´ ends. A C3 CPG spacer was added to the 3′ end of each blocking primer to prevent elongation. PCRs were carried out in a final volume of 25 μL, containing 2.5 μL of template DNA, 0.5 μM of the primers, 10 μM of each blocking primer, 12.5 μL of Supreme NZYTaq 2 × Green Master Mix (NZYTech), and ultrapure water up to 25 μL. The reaction mixture was incubated as follows: an initial denaturation step at 95 °C for 5 min, followed by 35 cycles (fungi) or 25 cycles (bacteria) of denaturing at 95 °C for 30 s, annealing at 62 °C for 45 s and 48 °C for 45 s (for fungi) or at 65 °C for 45 s and 50 °C for 45 s (for bacteria), extension at 72 °C for 30 s, and a final extension step at 72 °C for 10 min. The oligonucleotide indices that are required for multiplexing different libraries in the same sequencing pool were attached in a second PCR round with identical conditions but only 5 cycles and 60 °C as the annealing temperature.

The libraries were run on 2% agarose gels stained with GreenSafe (NZYTech) and imaged under UV light to verify the library size. Libraries were purified using Mag-Bind RXNPure Plus magnetic beads (Omega Biotek) following the manufacturer’s instructions. Root libraries were pooled together in equimolar amounts. Rhizosphere libraries were added to the pool, but the quantities were three times higher than those for the root libraries. The pool was sequenced in a fraction of a MiSeq PE300 run (Illumina).

### Bioinformatic analyses

We used QIIME2 software (Nov-2020 release) [30] to analyse the amplicon sequencing of the 16S rRNA gene and the ITS region. The raw paired reads were filtered and merged into amplicon sequence variants (ASVs) with the DADA2 plugin [31]. Forward and reverse reads were truncated at 299 nt and 250 nt, respectively, for 16S rRNA gene amplicons. The pairs of ITS sequences were truncated at 200 nt. We measured both alpha and beta diversity indices with QIIME2 plugins for statistical comparisons between categories of samples. We used the Kruskal‒Wallis test [32] to compare Shannon indices (alpha diversity) and permutation ANOVA (pseudo-F, 999 permutations) [33] to compare Bray‒Curtis distances (beta diversity), in both cases for categorical variables (all comparisons among (I) blueberry roots and (II) rhizosphere, and blackberry (III) roots and (IV) rhizosphere). We applied the Spearman test [34] to measure the correlation between continuous variables and Shannon index values. The quantification of the effect of metadata on the microbial compositions was measured through Adonis tests (999 permutations) [33, 35].

In the case of the bacterial communities, we assigned taxonomy to the ASVs based on the SILVA database (release 132–99%) [36]. Specifically, we extracted the database sequences based on the 16S rRNA primers detailed above and retained only the artificial amplicons with a size of 300–500 nt. Then, we trained the naïve Bayes classifier and classified the sequences using the scikit-learn plugin implemented in QIIME2. Similarly, we used the complete ITS sequences extracted from the UNITE database (v8, 04-Feb-2020) to classify the fungal sequences. We removed all the ASVs identified as mitochondrial or chloroplast genes in the 16 rRNA data.

We searched for specific enrichment or exclusion of microbial taxa over four categories: (i) roots vs. rhizospheres, (ii) blueberry vs. blackberry samples, (iii) blueberry roots vs. other samples, and (iv) blackberry roots vs. other samples. It has been proved that the use of the traditional *t*-test to study differential abundance of features or taxa led to high false-discovery rates (FDR) [37]. Hence, we decided to use the analysis of composition of microbiomes (ANCOM) framework [37] for these comparisons, which facilitates the search for differentially abundant features by considering compositional data. The results do not include *p-*values, but W statistic values, which represent the number of ANCOM subhypotheses that have passed for each taxa (from ASV to phyla), while always indicate, for significant values, that the relative abundance of a certain taxon is significantly different between groups (FDR-adjusted *p* < 0.05).

Taxa plots and boxplots were visualized with the ggplot2 (v3.3.2) package for R [38], and the UpSetR package (v1.4.0) [39] was employed to compare the presence/absence of taxa.

To inspect fungal trait abundances, we classified the identified genera in the ITS amplicon data into the categories described in the FungalTraits database (v1.2) [40]. Differences of abundance of fungal traits between sample types were analysed based on the relative abundance of each trait and applying a Tukey’s test for multiple comparisons (Tukey honestly significant difference [HSD]) in the analysis of variance (ANOVA) framework with the “stats” R package (v4.0.2).

## Results

### Microbial communities of wild blueberry and blackberry

We analysed the microbial communities of 42 roots and rhizospheres of wild blueberry and blackberry samples (Additional file 1: Fig. S1) through amplicon sequencing of the 16S rRNA gene and the ITS region amplicons. After quality filtering and the removal of plant 16S rRNA sequences, we obtained 1,674,878 final prokaryotic (mean: 22,855 for roots; 56,900 for rhizospheres) and 2,791,458 fungal (mean: 33,580 for roots; 99,352 for rhizospheres) paired sequences, which were sufficient to capture all the bacterial and fungal diversity of the samples according to the rarefaction curves (Additional file 2: Fig. S2). We grouped the all the sequences into a total of 24,598 16S rRNA ASVs and 12,373 ITS ASVs.

**Fig. 1:**
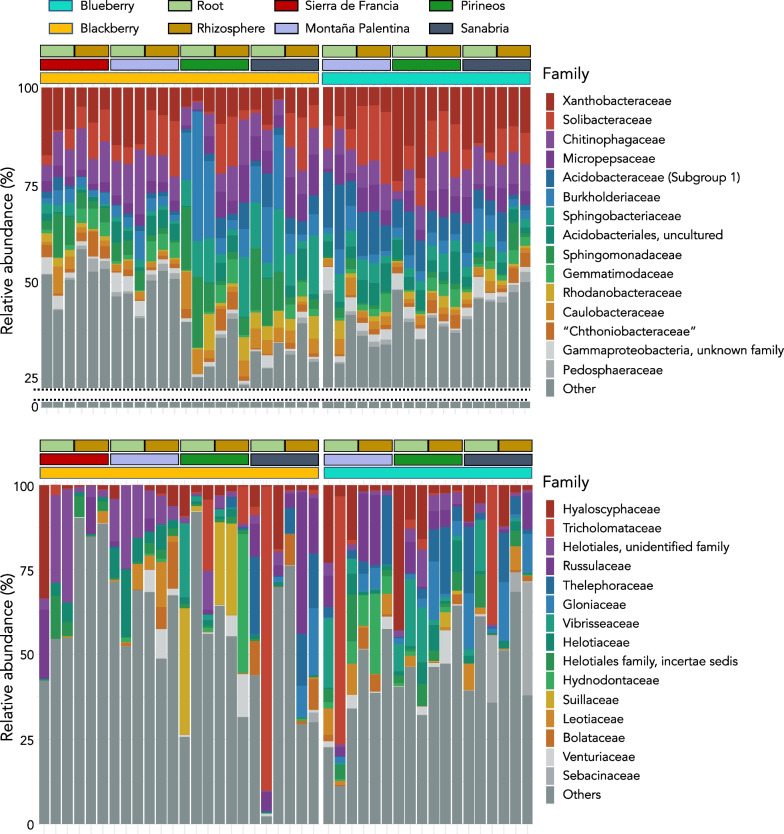
Taxa bar plot representing the relative abundance of the top 15 most abundant bacterial (top) and fungal (bottom) families within the analysed samples

The most abundant taxa in the root prokaryotic communities of both plants had roughly the same occurrence, and many of them had similar proportions. Proteobacteria was the main taxon in all roots (37.0–76.8%) and in some rhizosphere samples (32.3–50.7%). Acidobacteria was the second most abundant phylum in relative abundance (9.6–41.2% in rhizospheres; 0.8–26.7% in roots), followed by Bacteroidetes and Actinobacteria (Additional file 3: Fig. S3). In total, these communities harboured 36 different bacterial phyla, although a few (< 0.2%) archaeal sequences (4 phyla) were identified in some rhizospheric samples. Alphaproteobacteria was the most abundant bacterial class in all microbiomes, and Xanthomonadaceae (order Rhizobiales) was the most abundant family (Fig. 1). Following Xanthomonadaceae, the most representative families were Solibacteraceae (Acidobacteria), Chitinophagaceae (Bacteroidetes) and Micropepsaceae (Proteobacteria) (Fig. 1). Additionally, *Bradyrhizobium*, *Sphingomonas*, *Bryobacter*, *Mucilaginibacter*, *Granulicella*, *Acidibacter*, *Acidothermus* and *Rhodanobacter* were the most abundant identified genera in all the sample types.

In contrast, the fungal communities were less consistent among the different sample types and sampling sites (Fig. 1). For example, the abundance of Hyaloscyphaceae, which was the most abundant family, ranged from almost 40% of the reads in some samples but was absent or almost absent in others (Fig. 1). Ascomycota was the main phylum in blueberry roots, while there was more balance between Basidiomycota and Ascomycota in both plant rhizospheres (Additional file 3: Fig. S3). We also searched for ericoid mycorrhizal fungi (ErM)—mutualistic symbionts for Ericaceae plants such as *V. myrtillus*—and found several ErM-forming taxa within both blueberry and blackberry root and rhizospheric samples, such as 11 *Oidiodendron* species (e.g., *O. griseum*), 3 *Phialocephala* species (e.g., *P. fortinii*) and *Pezoloma ericae*.

Overall, along the different sampling locations, the bacterial composition of both blackberry and blueberry was shaped by the same most abundant families, being differentiated mainly by smooth shifts in their proportion (Fig. 1). Likewise, root and rhizosphere microbiomes were also distinguished by taxa abundance rather than by their occurrence (Fig. 1). However, in the case of the fungal communities, there is a higher dissimilarity of families among samples (Fig. 1).

**Fig. 2:**
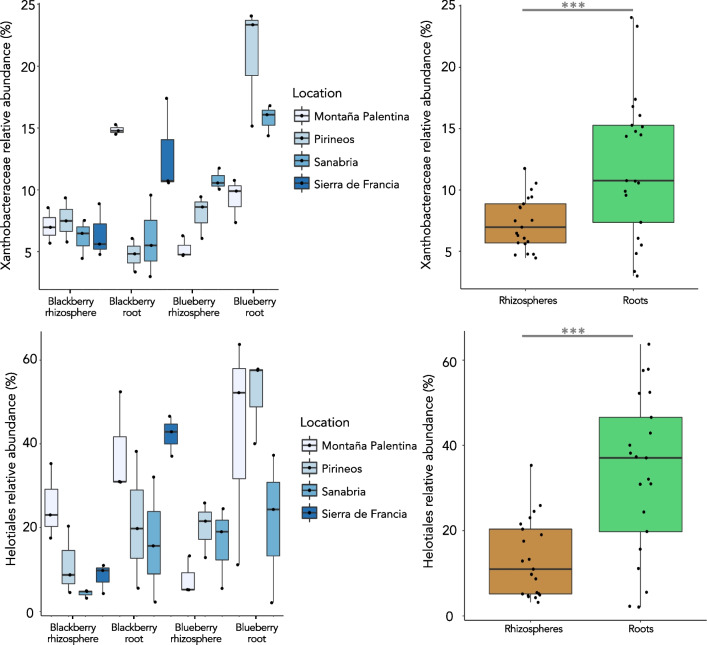
Boxplots representing the relative abundance of some distinctive microbial taxa within the studied samples grouped by location (left) and sample type (right). ***p ≤ 0.001

The roots of both plants showed an enrichment of Proteobacteria; in contrast, Gemmamonidetes was enriched in both rhizospheres (Additional file 4: Fig. S4). Interestingly, Acidobacteria were found in similar proportions in both blueberry roots and rhizospheres but the same was not true in blackberry, where it seemed to be more excluded from roots, while Actinobacteria was enriched in blueberry roots (Additional file 4: Fig. S4). Regarding the bacterial composition of blackberry roots, there was an increase in the abundance of Sphingobacteriaceae, Sphingomonadaceae and Burkholderiaceae (Fig. 1). Interestingly, we found noteworthy changes in the main proportions of the most abundant bacterial family in our data, Xanthobacteraceae. This taxon was more abundant in roots than in the rhizosphere (ANOVA, p = 0.001) (Fig. 2). However, the plant species and sampling site affected its selective enrichment, as it was more abundant in blueberry plants mostly from the Pyrenees (Fig. 2).

Conversely, the abundance of the main fungal order of the studied communities, Helotiales, which comprises ErM-forming taxa, was also significantly more abundant in the root endospheres (ANOVA, p < 0.001) (Figs. 1, 2).

### Factors shaping microbial community assembly

**Fig. 3:**
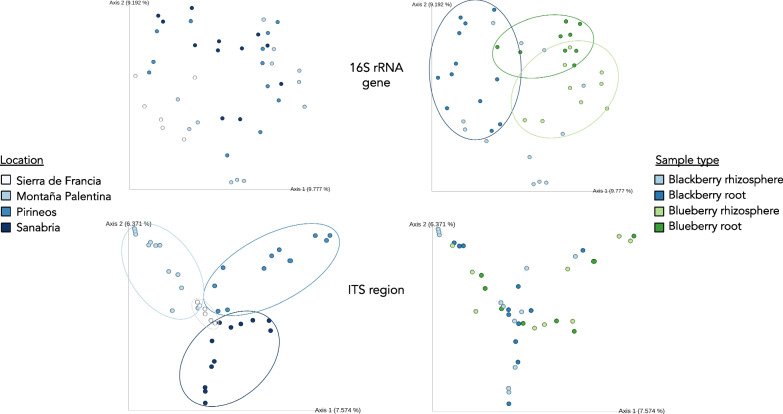
PCoA of the bacterial (top) and fungal (bottom) communities. The bacterial communities are easily clustered by plant species and sample type, while for fungi, the main predictor of diversity was location

We used both alpha- and beta-diversity indices to depict the similarity between and among sample types. There were significant differences in the bacterial composition (Bray–Curtis index) between different plant species, plant tissues, and locations (permutation ANOVA, *p* values ≤ 0.01). Samples from the same plant species and tissues were easily clustered in the PCoA (Fig. 3). In contrast, the fungal communities showed more differentiated clustering based on location, and only within each location were samples slightly clustered by plant species (permutation ANOVA, *p* values ≤ 0.01) (Fig. 3). However, the fungal assemblages of each plant root and rhizosphere were not significantly different (Additional file 7: Table S1). We measured the contribution of each variable to the microbial assemblage through Adonis analyses. Although location was the main factor that influenced both the bacterial and fungal communities (R^2^ = 0.17; Pr(> F) = 0.001), both variables—plant species and sample type (root vs. rhizosphere)—affected the bacterial (R^2^ = 0.07; Pr(> F) = 0.001) more than the fungal (R^2^ = 0.045 and 0.03; Pr(> F) = 0.001 and 0.004, respectively) composition (Fig. 3).

We also studied the dependence of the alpha diversity on continuous variables, but in our data, neither the pH values nor the elevation above sea level of the sites were significantly correlated with the microbial diversity (*p* values > 0.1).

### Roots act as selective barriers for bacterial endophytes

**Fig. 4:**
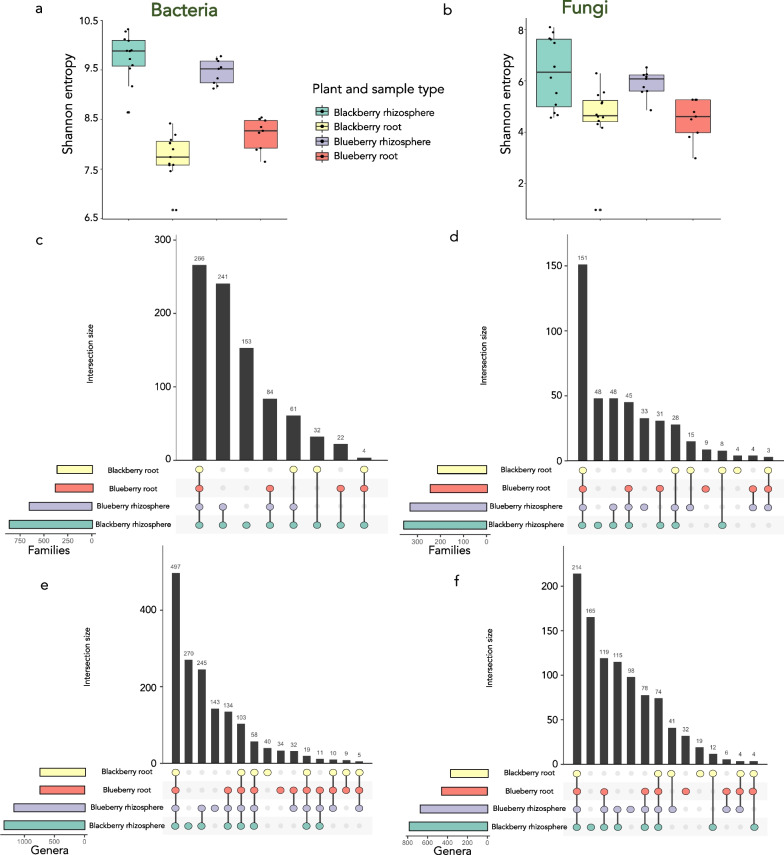
Alpha diversity measurements (Shannon index) of the blackberry and blueberry root bacterial (**a**) and fungal (**b**) communities. **c**, **d** UpSetR graph depicting the occurrence of unique and shared bacterial (**c**) and fungal (**d**) families within each plant tissue type and plant species. **e**, **f** UpSetR-graph depicting the occurrence of unique and shared bacterial (**e**) and fungal (**f**) genera within each plant tissue type and plant species

The plant root endophytic microbiome usually harbours specialized microbes that share a close relationship with the plant. There should be mutualistic selection for the enrichment of certain microorganisms in these tissues. Here, we found that the root barrier is a main factor preventing entrance to many taxa, and therefore, the endophytic diversity was lower (Fig. 4). However, we found that this selection was less considerable in fungi. Blueberry roots harbor a higher diversity of bacterial taxa than blackberry roots (Kruskal‒Wallis, H = 5.5, *p*-value = 0.02) (Fig. 4). This finding is contrary to the bacterial diversity found in rhizospheres, where blackberry attracted a significantly higher bacterial diversity than blueberry (Kruskal‒Wallis, H = 3.68, *p* value = 0.05) (Fig. 4). These trends agree with the beta diversity (Bray–Curtis) distances mentioned above (Additional file 7: Table S1).

**Fig. 5:**
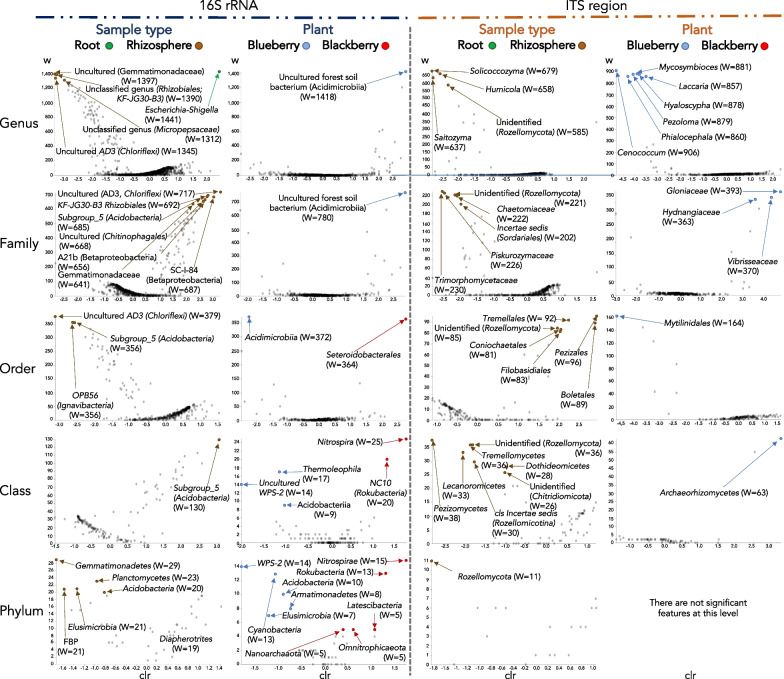
Taxa significantly enriched in each plant species or sample type. Analysis performed with ANCOM. W statistic values represent the number of ANCOM subhypotheses that have passed for each taxa (FDR-adjusted p < 0.05). *clr* centered logarithmic transforms

In contrast, we searched for significant enrichments or exclusions of microbial taxa in plant roots, considering all the different samples from this study. We found many microbial taxa significantly associated with rhizospheres, but only one taxon with significant enrichment in the endosphere, an Enterobacteriaceae genus (‘*Escherichia*–*Shigella’*) (W = 1,441) (Fig. 5, top left panel).

### Some microbes show specificity towards one plant species

Some microbial taxa appeared to be more enriched in samples from one plant than in those from the other (Fig. 5). For instance, Acidimicrobiia (bacterial order), several bacterial phyla (e.g., Acidobacteria, Cyanobacteria, WPS-2), and several fungi, such as the genera *Cenococcum*, *Pezoloma*, *Mycosymbioces*, *Laccaria*, *Phialocephala* and *Hyaloscypha*, and the class Archaeorhizomycetes were more abundant in blueberry plants. In contrast, several bacterial taxa were more enriched in blackberry plants, such as the order Steroidobacterales and the phyla Nitrospirae and Rokubacteria (Fig. 5). Interestingly, we detected a nanoarchaeal phylum (Nanoarchaeota) of the DPANN superphylum, which was more abundant in blackberries (Fig. 5).

**Fig. 6:**
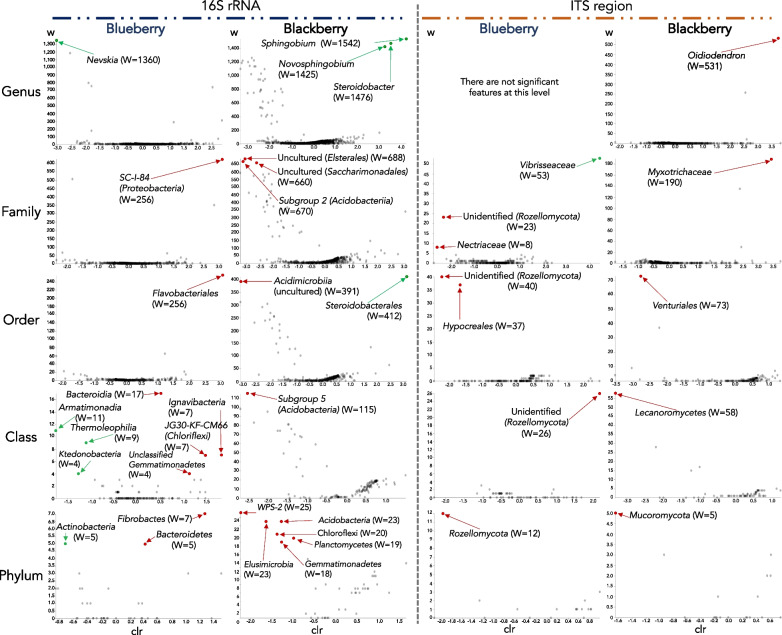
Taxa significantly enriched (green) or excluded (red) in the roots of each plant species. Analysis performed with ANCOM. W statistic values represent the number of ANCOM subhypotheses that have passed for each taxa (FDR-adjusted p < 0.05). *clr* centered logarithmic transforms

Considering the specific and intimate plant‒microbe interactions that may occur within the plant endosphere, we also searched for microbial specificity towards the roots of each plant. We found that both blueberry and blackberry roots significantly excluded many diverse microbial taxa, and only 4 genera were significantly enriched in these roots: *Nevskia* in blueberry roots and *Novosphingobium*, *Sphingobium* and *Steroidobacter* in blackberry roots (Fig. 6, Additional file 5: Fig. S5). Additionally, the fungal family Vibrisseaceae was significantly enriched in blueberry roots. At higher taxonomic levels, we found that the classes Armatimonadia, Thermoleophilia and Ktedonobacteria and the phylum Actinobacteria were significantly associated with the blueberry roots, and the order Steroidobacterales was significantly associated with blackberry roots (Fig. 6).

### Differential abundance of fungal traits

**Fig. 7:**
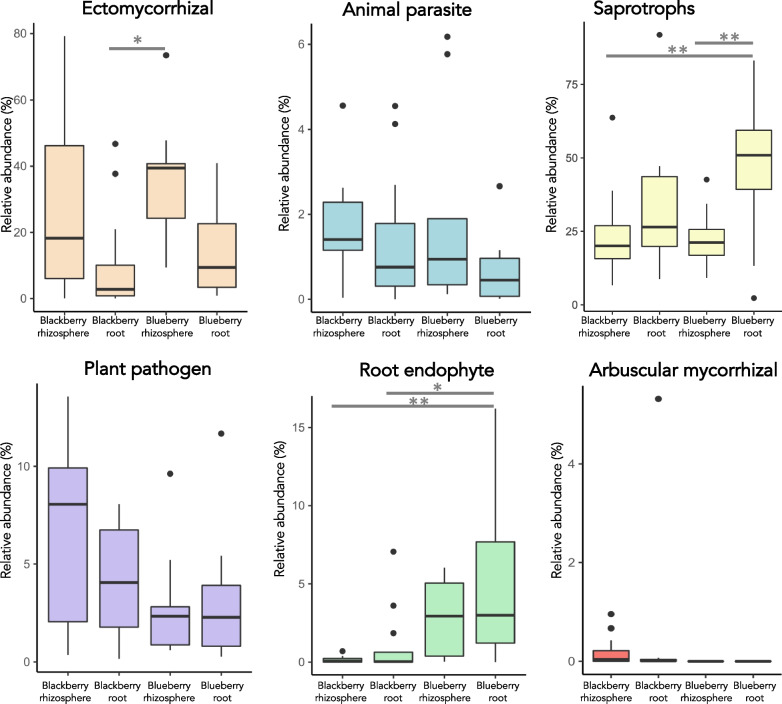
Differential abundance of fungal traits among the different sample categories. *p-adj < 0.05; **p-adj < 0.01

Some fungal taxa are specialized into certain lifestyles depending on their nutritional mode, host range, or beneficial pathogenic behaviour within hosts, among other factors. Here, we classified the fungi into the categories available on the FungalTraits database [40]. A total of 729 fungal genera, out of the 981 present among the different samples, were classified within some fungal trait. Among the traits with higher abundance in the samples, those classified as saprotrophs were significantly more enriched in blackberry roots than in both plant rhizospheres (p-adj < 0.01) (Fig. 7). We split the saprotroph category into subcategories, and found that this dissimilarity was due mainly to the differential abundance of soil and litter saprotrophs ( Additional file 6: Fig. S6). In addition to saprotrophs, the other traits showed some disparities between sample types, but not with enough statistical significance. Only the fungi classified as root endophytes were significantly more enriched in blueberry roots than in blackberry rhizospheres (p-adj < 0.01) and roots (p-adj < 0.05), and ectomycorrhizal fungi abundances were significantly different between blueberry rhizospheres and blackberry roots (p-adj < 0.05) (Fig. 7).

## Discussion

Here, we present new data that help to understand microbe‒plant associations and the factors that affect plant microbial communities in wild ecosystems. We provide relevant data to understand the microbial fluxes within and between both the rhizospheres and root endospheres of two distant plant species.

Some microbial taxa may inhabit the plant environment transiently or reflect a stochastic occurrence [41]. Nonetheless, we found a similar occurrence pattern of the main fungal and bacterial taxa in taxonomically distant plants, which supports the association of those microbes with the plant niche, independent of the plant species. Additionally, some plants can also establish very specific symbiosis with some microbial species; to further investigate which microbes have a unique association with each of the two plant species studied, we searched for enriched microbes in the roots (endosphere) of each species. We found significant enrichment of *Nevskia* sequences in blueberry roots and of *Sphingobium*, *Novosphingobium* and *Steroidobacter* in blackberry roots. We argue that strains belonging to these genera may have coevolved with their respective plants, although further research should be performed to demonstrate their beneficial roles for both hosts. Although the presence of *Nevskia* in soils is well documented [42–45], no relationship between species of this genus and blueberry plants or any other plant has been suggested. Similarly, *Sphingobium*, *Novosphingobium* and *Steroidobacter* have not been isolated before from blackberry, and only *Steroidobacter* OTUs have been detected in its relative raspberry (*Rubus idaeus*) [46].

It has been proposed that the fungal composition of plant microbiomes is mainly affected by environmental conditions (e.g., temperature, rainfall, C/N ratio) rather than by the plant species, and the opposite has been proposed for bacteria [6, 17–19]. Nevertheless, we found that location was the main factor that explained both the bacterial and fungal composition. However, we found a clear clustering of bacterial communities that separated sample groups by plant and sample type. Considering the differences between sample types, we found that the bacterial communities of both blackberry and blueberry roots were significantly different from those in the rhizosphere but not in the case of the fungal communities. This could be explained by the extension of the ericoid mycorrhizae beyond the root area.

Our results showed that plant species affected the bacterial composition of both plants, but interestingly, the mean proportion of the most abundant taxa was similar between blackberry and blueberry. At the phylum level, our data agreed with those obtained from many distant plants [3, 8, 47], with the exception of Acidobacteria. According to the literature, this phylum is usually more abundant in rhizospheres than in roots, including in *Vaccinium angustifolium* plants [24]; nonetheless, this was not the case for our *V. myrtillus* samples, where we found a higher abundance in root tissues. Considering that blueberry inhabits acidic soils [48], the abundance of Acidobacteria in endospheric tissues may not only be stochastic but also reflect some dependence on this acidophilic bacterial phylum.

In contrast, Helotiales was enriched in blueberry roots. This fungal order comprises many ericoid mycorrhizae that have been largely associated with *Vaccinium* plants [49, 50]. We found that some of the most abundant ericoid mycorrhizal species in blueberry roots were *Pezoloma ericae*, *Phialocephala fortinii* and *Oidiodendron* spp., which supports previous reports on these plant communities [24, 51]. Many of the fungal taxa detected in our analyses agreed with the rhizosphere fungal composition of *V. angustifolium*, but while Yurgel et al. [24] found Lipomycetaceae to be an abundant family, it was almost absent in *V. myrtillus*, which suggests that it is a transient family or that it has some level of specificity with *V. angustifolium*.

Finally, we found a dissimilar fungal trait distribution in the studied microbiomes. Specifically, saprotrophs were especially enriched in blueberry roots. This result does not agree with previous reports since it is usually found that these generalist fungi usually inhabit bulk soils, where they recycle dead biomass [52, 53]. Their enrichment in blueberry roots may be related to some recycling of host cell material, but further research is needed to understand why this microbial shift occurs specifically within the blueberry endosphere.

In summary, our data demonstrate new microbe‒plant associations in wild systems. We also found a stronger filtering and selection of bacteria by roots than that applied to fungi. Furthermore, we present the first insights into the microbiome composition of blackberry. Overall, our results help to understand the microbiology of forests and, particularly, to understand specific plant‒microbe associations in natural environments.

## Supplementary Information

**Additional file 1 **Schematic description of the sampling locations and sample types used in this study. Red dots are locations where both blueberry and blackberry plants were sampled. The orange dot represents a location where only wild blackberry plants were collected. The background map was obtained from https://www.d-maps.com/conditions.php?lang=es

**Additional file 2 **Alpha rarefaction curves of the alpha diversity (Shannon index) of the 16S rRNA gene (top) and ITS region (bottom) metabarcoding samples grouped by sample categories. The X-axis represents the number of reads

**Additional file 3 **Taxa bar plot representing the relative abundance of the bacterial (top) and fungal (bottom) phyla within the analysed samples

**Additional file 4 **Boxplots representing the relative abundance of some distinctive microbial taxa within the studied samples grouped by plant species and sample type

**Additional file 5 **Boxplots representing the relative abundance of bacterial genera found to be significantly enriched in the roots of blueberry (*Nevskia*) or blackberry (*Sphingobium*, *Novosphingobium *and *Steroidobacter*) within the studied samples grouped by plant species and sample type

**Additional file 6 **Differential abundance of saprotrophic fungal traits among the different sample categories

**Additional file 7 **Results for the comparison (permutation ANOVA) of beta-diversity indices (Bray–Curtis) of the different plant species and sample types (root and rhizosphere)

## Data Availability

All raw sequences obtained in this study have been deposited in the NCBI Sequence Read Archive (SRA) database under BioProject PRJNA942612. The bioinformatics codes and source data can be accessed in the following GitHub Repository: https://github.com/zakisaati/blueberry_blackberry_microbiomes.
