# Supplementary material for: Microbiome specificity and fluxes between two distant plant taxa in Iberian forests
Source: Environ Microbiome. 2023 Jul 22;18:64. doi: 10.1186/s40793-023-00520-x (PMC10363313; doi:10.1186/s40793-023-00520-x)
Supplement: Supplementary file 1 — Additional file 1 Schematic description of the sampling locations and sample types used in this study. Red dots are locations where both blueberry and blackberry plants were sampled. The orange dot represents a location where only wild blackberry plants were collected. The background map was obtained from https://www.d-maps.com/conditions.php?lang=es [file 40793_2023_520_MOESM1_ESM.pdf]

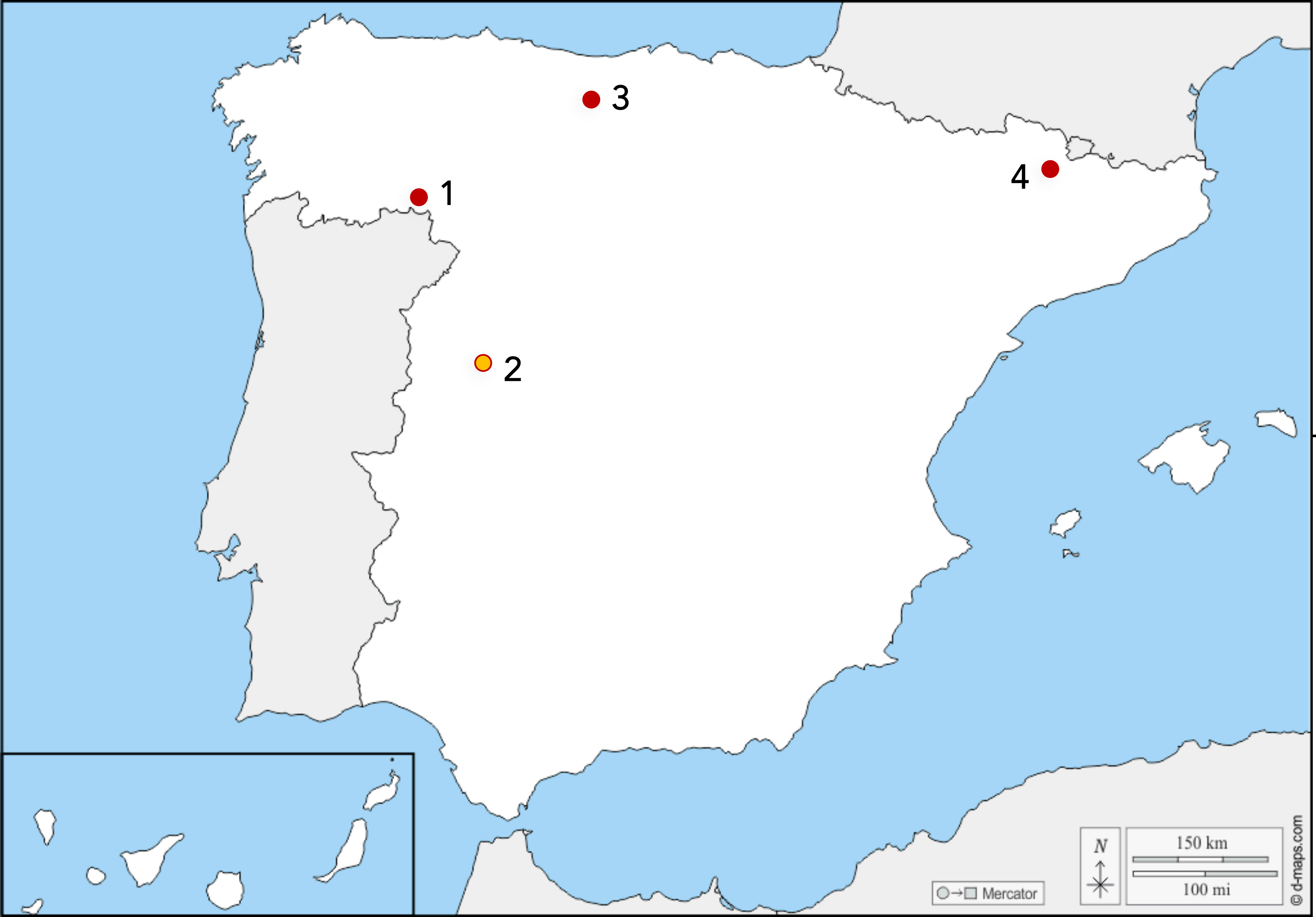

Samples	Blueberry	Blackberry	Total
Root	9	12	21
Rhizosphere	9	12	21
Total	18	24	**42**

Target DNA

- ITS region: 42 sequencing samples
- 16S rRNA: 42 sequencing samples
- Total: **84 sequencing samples**

1- Sotillo de **Sanabria**, Parque Natural del Lago de Sanabria, Zamora. 42.0838061N / 6.7295119W. Soil pH = 5.2. Elevation: 1125 m

2- Linares de Riofrío, **Sierra de Francia**, Salamanca. 40.5787884N / 5.9411208 W. Soil pH = 5.8. Elevation: 1015 m

3- Puerto de Piedrasluengas, **Montaña Palentina**, Palencia-Santander. 43.0411667N / 4.4597778W. Soil pH = 5.0. Elevation: 1355 m

4- Sant Joan de l´Erm, Parque Nacional de los **Pirineos**, Lleida. 42.4172981N / 1.2876350W. Soil pH = 5.4. Elevation: 1726 m
