# Supplementary figures and images for: Microbiome specificity and fluxes between two distant plant taxa in Iberian forests
Source: Environ Microbiome. 2023 Jul 22;18:64. doi: 10.1186/s40793-023-00520-x (PMC10363313; doi:10.1186/s40793-023-00520-x)

# 16S rRNA

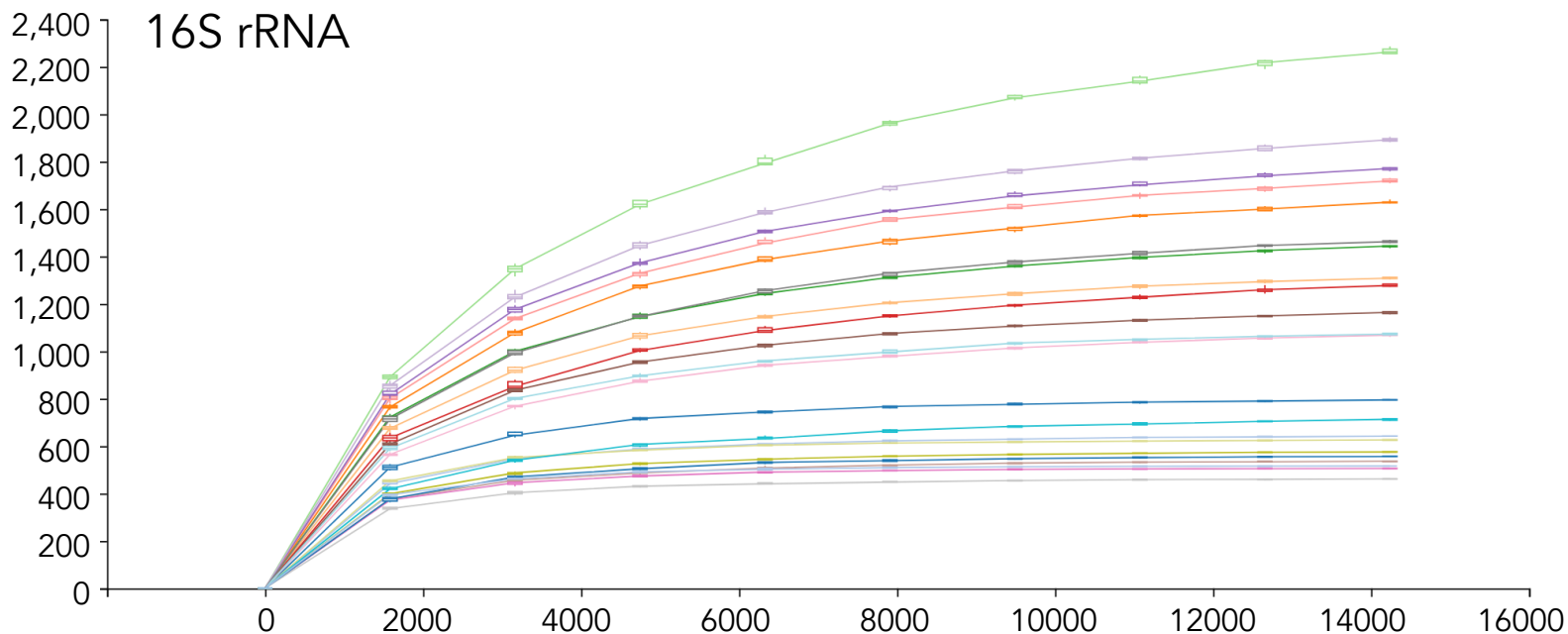

# ITS

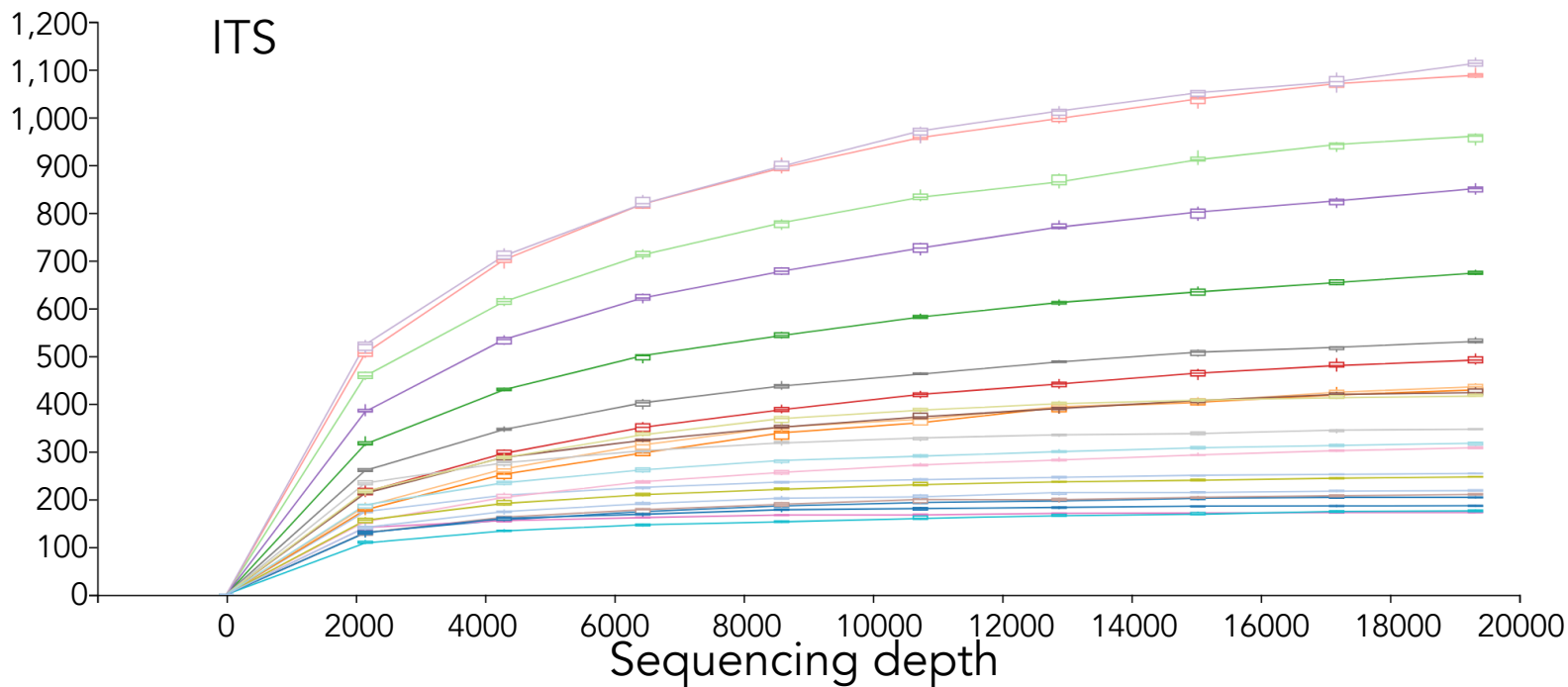

Supplement: Supplementary file 2 — Additional file 2 Alpha rarefaction curves of the alpha diversity (Shannon index) of the 16S rRNA gene (top) and ITS region (bottom) metabarcoding samples grouped by sample categories. The X-axis represents the number of reads [file 40793_2023_520_MOESM2_ESM.pdf]

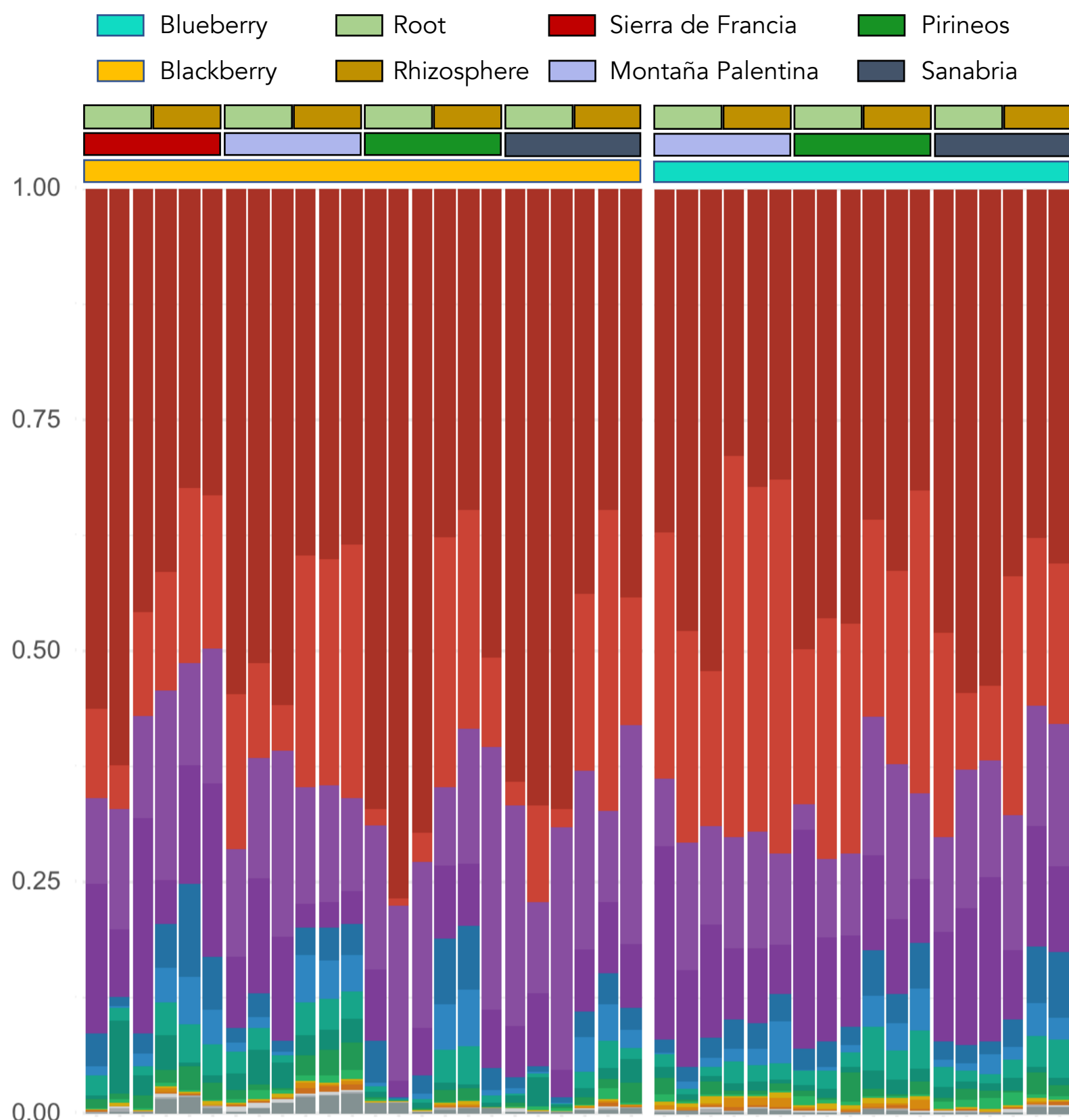

## Phyla

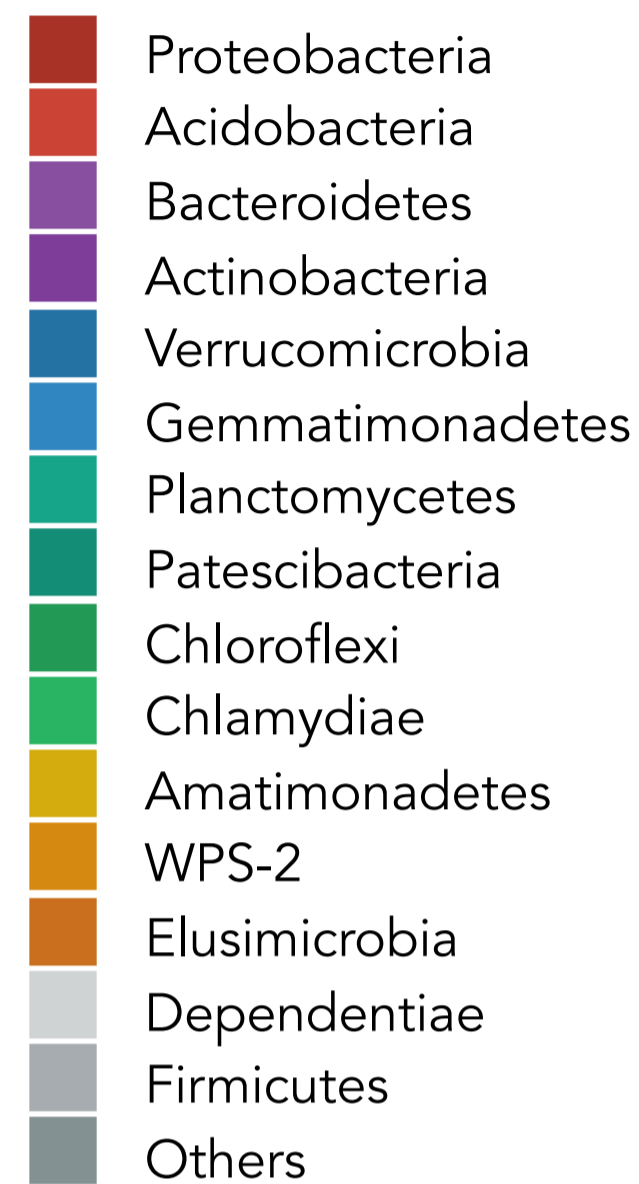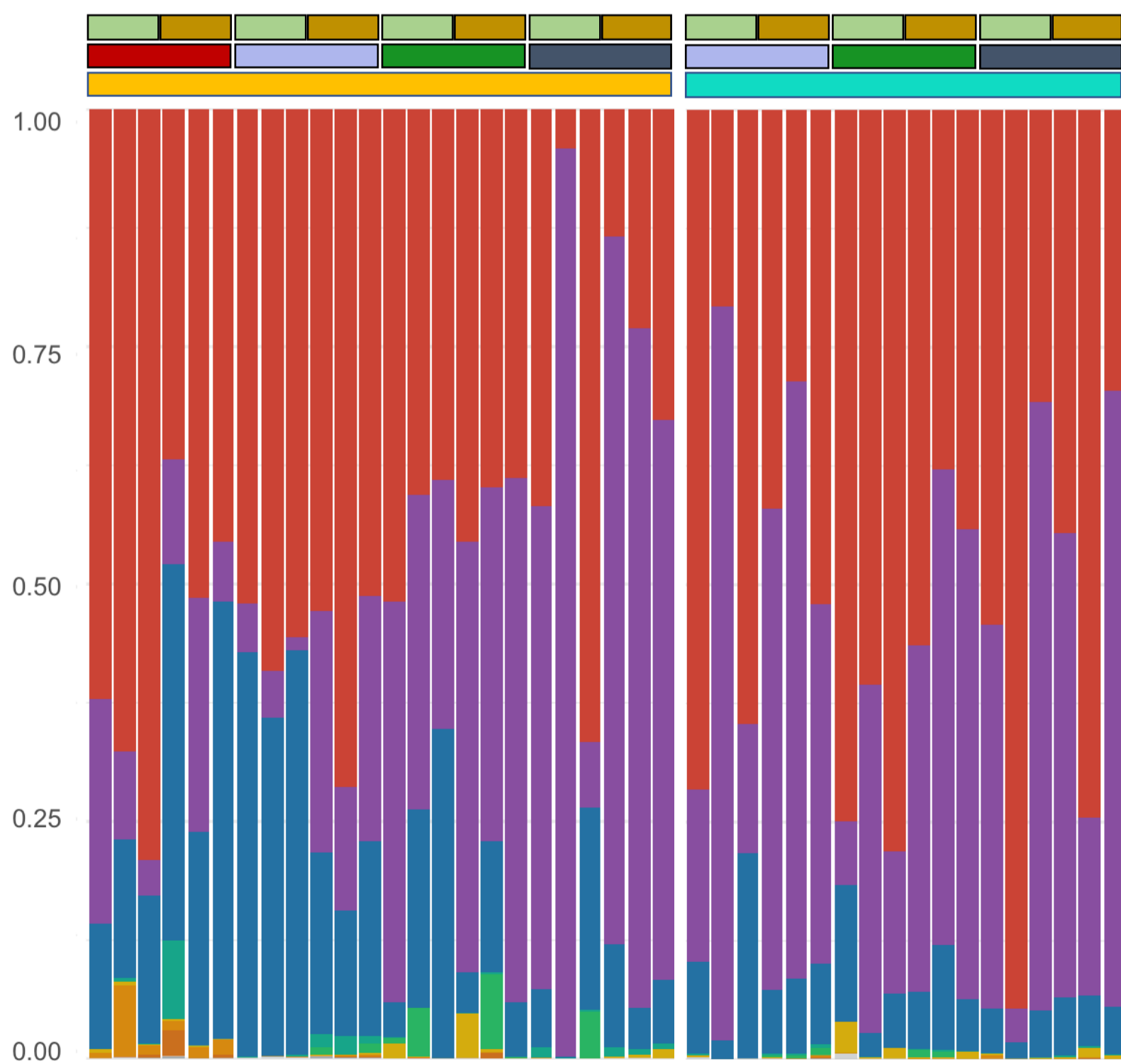

## Phyla

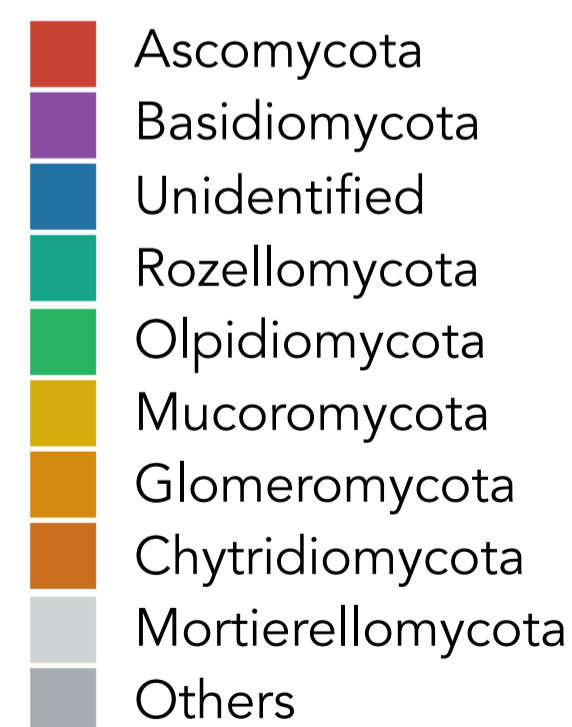

Supplement: Supplementary file 3 — Additional file 3 Taxa bar plot representing the relative abundance of the bacterial (top) and fungal (bottom) phyla within the analysed samples [file 40793_2023_520_MOESM3_ESM.pdf]

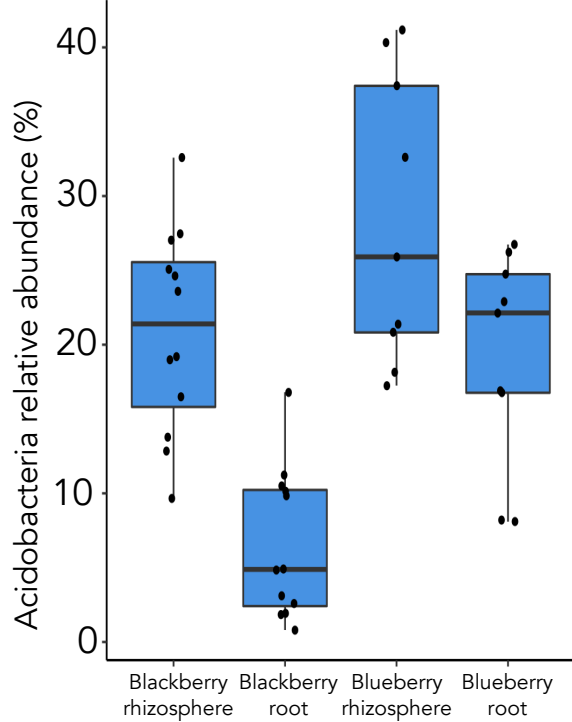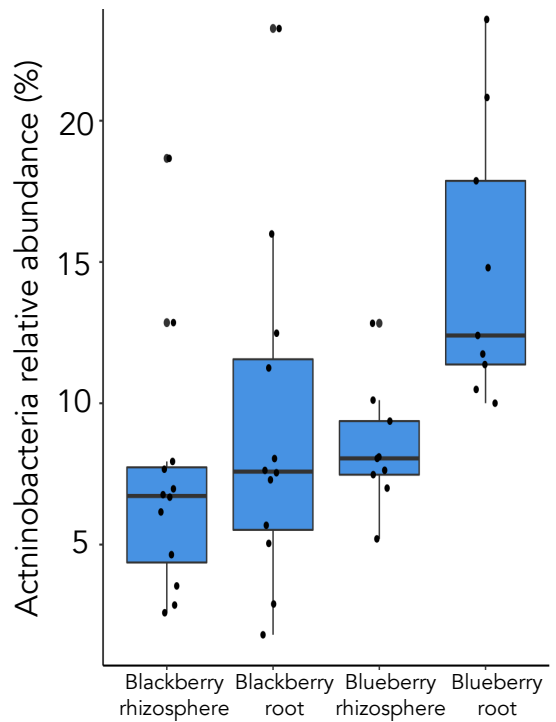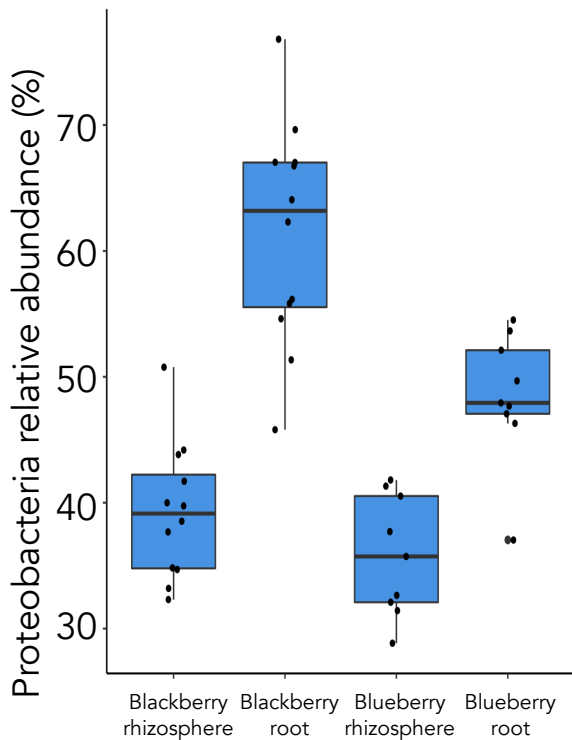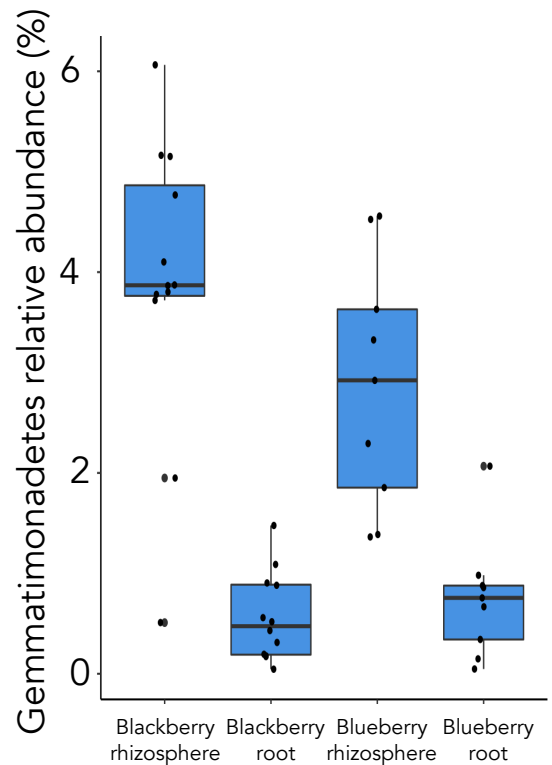

Supplement: Supplementary file 4 — Additional file 4 Boxplots representing the relative abundance of some distinctive microbial taxa within the studied samples grouped by plant species and sample type [file 40793_2023_520_MOESM4_ESM.pdf]
