# Supplementary material for: Microbiome specificity and fluxes between two distant plant taxa in Iberian forests
Source: Environ Microbiome. 2023 Jul 22;18:64. doi: 10.1186/s40793-023-00520-x (PMC10363313; doi:10.1186/s40793-023-00520-x)
Supplement: Supplementary file 5 — Additional file 5 Boxplots representing the relative abundance of bacterial genera found to be significantly enriched in the roots of blueberry (Nevskia) or blackberry (Sphingobium, Novosphingobium and Steroidobacter) within the studied samples grouped by plant species and sample type [file 40793_2023_520_MOESM5_ESM.pdf]

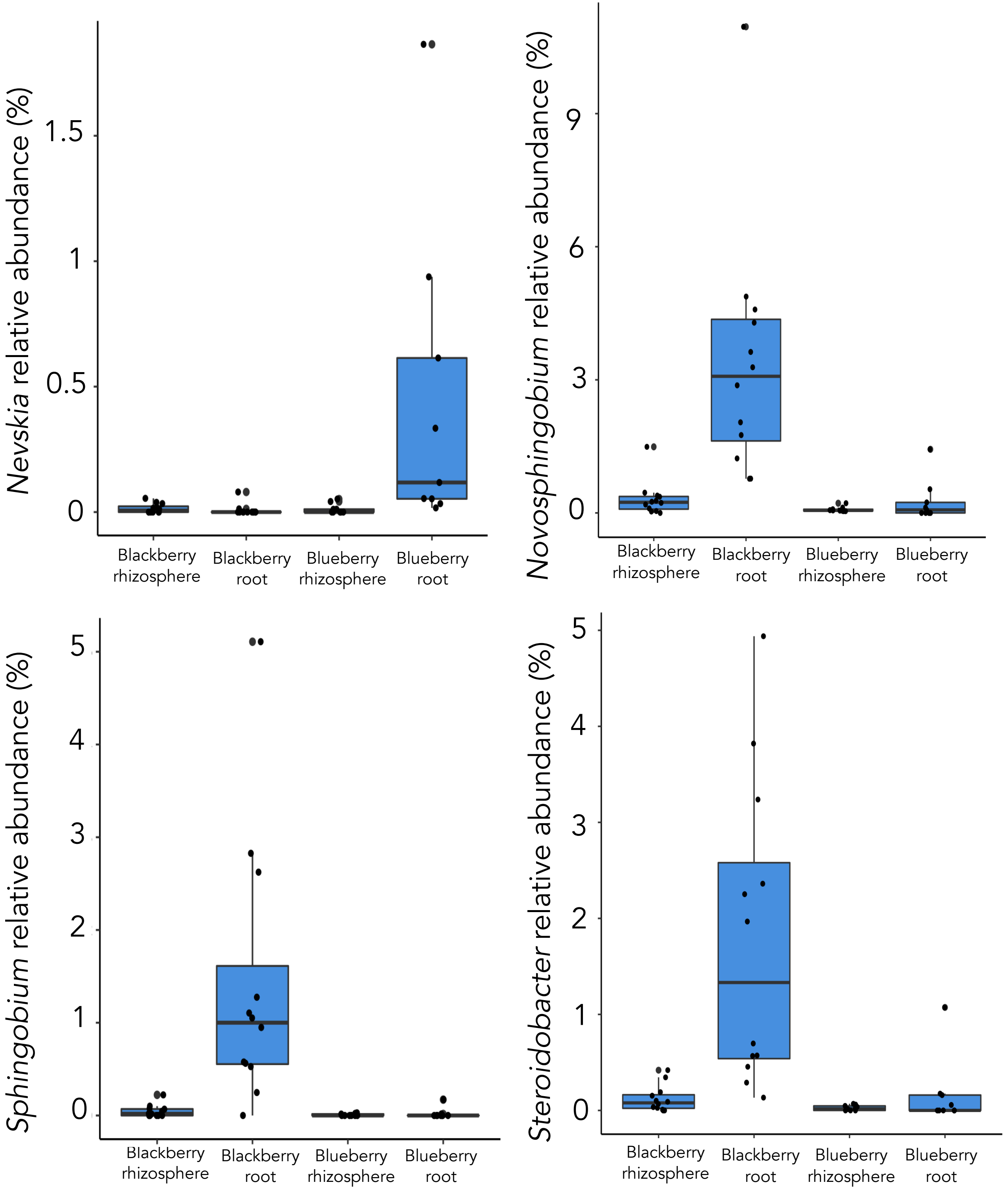

Nevskia relative abundance (%)
0
0.5
1
1.5
Blackberry rhizosphere
Blackberry root
Blueberry rhizosphere
Blueberry root
Novosphingobium relative abundance (%)
0
3
6
9
Blackberry rhizosphere
Blackberry root
Blueberry rhizosphere
Blueberry root
Sphingobium relative abundance (%)
0
1
2
3
4
5
Blackberry rhizosphere
Blackberry root
Blueberry rhizosphere
Blueberry root
Steroidobacter relative abundance (%)
0
1
2
3
4
5
Blackberry rhizosphere
Blackberry root
Blueberry rhizosphere
Blueberry root
