# Supplementary material for: Microbiome specificity and fluxes between two distant plant taxa in Iberian forests
Source: Environ Microbiome. 2023 Jul 22;18:64. doi: 10.1186/s40793-023-00520-x (PMC10363313; doi:10.1186/s40793-023-00520-x)
Supplement: Supplementary file 6 — Additional file 6 Differential abundance of saprotrophic fungal traits among the different sample categories [file 40793_2023_520_MOESM6_ESM.pdf]

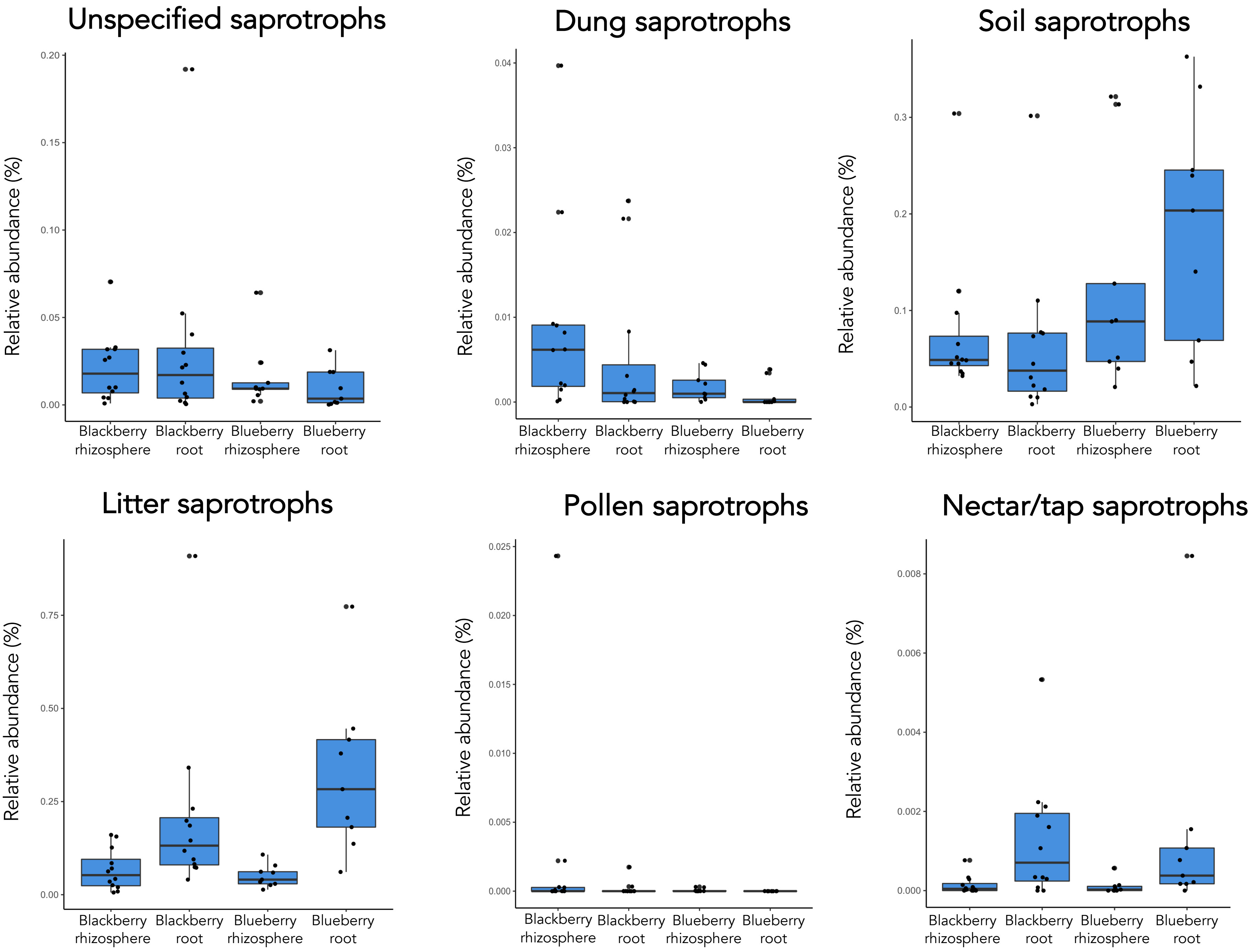
Unspecified saprotrophs
Dung saprotrophs
Soil saprotrophs
Litter saprotrophs
Pollen saprotrophs
Nectar/tap saprotrophs
Relative abundance (%)
Blackberry rhizosphere
Blackberry root
Blueberry rhizosphere
Blueberry root
0.00
0.05
0.10
0.15
0.20
0.01
0.02
0.03
0.04
0.0
0.1
0.2
0.3
0.25
0.50
0.75
0.000
0.005
0.010
0.015
0.020
0.025
0.002
0.004
0.006
0.008
