# Supplementary material for: Microbiome specificity and fluxes between two distant plant taxa in Iberian forests
Source: Environ Microbiome. 2023 Jul 22;18:64. doi: 10.1186/s40793-023-00520-x (PMC10363313; doi:10.1186/s40793-023-00520-x)
Supplement: Supplementary file 7 — Additional file 7 Results for the comparison (permutation ANOVA) of beta-diversity indices (Bray–Curtis) of the different plant species and sample types (root and rhizosphere) [file 40793_2023_520_MOESM7_ESM.docx]

**Supplementary Table 1.** Results for the comparison (permutation ANOVA) of beta-diversity indices (Bray–Curtis) of the different plant species and sample types (root and rhizosphere).

|  |  | **ITS** | | **16s RNA** | |
| --- | --- | --- | --- | --- | --- |
| **Group 1** | **Group 2** | **pseudo-F** | **q-value** | **pseudo-F** | **q-value** |
| Blackberry rhizosphere | Blackberry root | 1.09 | 0.320 | 2.46 | 0.001 |
|  | Blueberry rhizosphere | 1.45 | 0.035 | 1.82 | 0.002 |
|  | Blueberry root | 1.67 | 0.003 | 3.17 | 0.001 |
| Blackberry root | Blueberry rhizosphere | 1.67 | 0.003 | 3.22 | 0.001 |
|  | Blueberry root | 1.45 | 0.008 | 2.74 | 0.001 |
| Blueberry rhizosphere | Blueberry root | 0.98 | 0.48 | 2.10 | 0.004 |
